# Comparative *in vitro* study on biomechanical behavior of zirconia and polyetheretherketone biomaterials exposed to experimental loading conditions in a prototypal simulator

**DOI:** 10.7150/ijms.82297

**Published:** 2023-04-02

**Authors:** Vincenzo Vertucci, Benedetta Marrelli, Roberta Ruggiero, Mario Iaquinta, Gaetano Marenzi, Gian Marco Parisi, Roberta Gasparro, Andrea Pacifici, Gianfranco Palumbo, Gilberto Sammartino, Marco Tatullo

**Affiliations:** 1 Tecnologica Research Institute - Marrelli Health, 88900 Crotone, Italy; 2 Department of Neurosciences, Reproductive and Odontostomatological Sciences, Postgraduate School of Oral Surgery, University “Federico II” of Naples, via S. Pansini 5, 80131 Naples, Italy; 3 Dentist in Rome, 00195 Rome, Italy; 4 Department of Oral and Maxillo-Facial Sciences, Sapienza University of Rome, 00195 Rome, Italy; 5 Department of Mechanics Management and Mathematics (DMMM), Politecnico di Bari, Viale Japigia, 182, 70126 Bari, Italy; 6 Department of Translational Biomedicine and Neurosciences (DiBraiN), University of Bari ALDO MORO, 70124 Bari, Italy; 7 Honorary Senior Clinical Lecturer— University of Dundee, Dundee, Scotland DD1 4HR, UK; 8 Founder Member of MIRROR—Medical Institute for Regeneration and Repairing and Organ Replacement, Interdepartmental Center, University of Bari ALDO MORO, 70124 Bari, Italy

**Keywords:** Zirconia, PEEK, biomaterials, three-point bending test, FPDs

## Abstract

Zirconia and polyetheretherketone (PEEK) are two biomaterials widely investigated as substitute for metals in oral prosthetic rehabilitation. To achieve a proper biomechanical behavior, the prosthetic biomaterials must ensure a good resistance to loads, as this is a crucial characteristic enabling their use in dental applications. The aim of this study was to investigate differences in the fracture resistance of different biomaterials in an experimental environment: fixed partial dentures (FPDs) screwed in a prototype of biomimetic mandible. 10 Samples of FPDs were allocated in 2 groups (A and B): Group A (n=5) involved FPDs in zirconia-ceramic, and Group B (n=5) involved FPDs in PEEK-composite. The samples were loaded by means of a three-point bending mechanical test, and the load to fracture has been evaluated generating a point-by-point graphics (speed/load and time/deformation). The samples were further analyzed by micro-computed tomography (micro-CT) and described under experimental loading conditions. Zirconia-ceramic FDPs were the samples reporting the worst results, showing a lower value of vertical displacement with respect to PEEK-based samples. The micro-CT results have further confirmed the preliminary results previously described. This *in vitro* study aims to give analytic data on the reliability of PEEK as a reliable and strong biomaterial for prosthetic treatments.

## Introduction

In oral rehabilitation ceramics are useful for replacing implants, dental bridges, crowns, artificial denture teeth with the aim to improve aesthetic characteristics and mimic the natural teeth 1-4. Different biomaterials are proposed in regenerative dentistry with different approaches, cell-based and cell-free approaches 5-8. The main advantages of dental ceramics are color stability, low thermal conductivity, biocompatibility, high wear resistance; these attractive properties explain their use in dentistry 9-11. Additionally, some improvements in their composition have been reported in literature 12-14. Ceramics have defects that become fractures whenever the deformation is more than 0.1-0.3% 15. Zirconia biomaterials are among the most popular ceramic materials for dental applications 16-20. Zirconia is a crystalline dioxide of zirconium (ZrO_2_) owning metals-like mechanical properties. Following a stress on ZrO_2_ surface, compression resistance of ZrO_2_ is approximately 2000 MPa) 21. Pure Zirconia is monoclinic (m) at room temperature and pression. With the increase in temperature, Zirconia shows various polymorphism, by passing from tetragonal to a cubic structure 22. These transformations are martensitic: it represents a phase change obtained by a crystal structure at the solid state. Zirconia is the main ceramic system able to show significant transformation 23.

In addition to ceramics, also polymers are new materials used to fabricate dental frameworks. Among these, polyetheretherketone (PEEK) is the most used polymer in dentistry: it is part of the polyaryletherketone (PAEK) family to which also belongs another polymeric material, the polyetherketoneketone (PEKK) 24. PEEK has a low elastic modulus, high fracture strength, dimensional stability, and a good biocompatibility; these properties make it a substitute to conventional biomaterials for dental application 25. However, few studies are available on fixed partial dentures (FDPs) 26.

Although ceramics and dental restorative compounds have elastic properties and biocompatibility, they are susceptible to brittle fractures. Brittle materials are characterized by little deformation, poor capacity to resist impact and vibration of load, high compressive strength, and low tensile strength. 32.

With these premises, the aim of this study was to evaluate the fracture strengths in both fixed partial dentures of zirconia-ceramic and PEEK-composite: such analysis has been carried out on a case-study with two fixtures screwed in an experimental resin mandible. This experimental mandible is technically a “mandibular section simulator” (MSS) reproducing the human mandible limitedly to the premolar and molar area: the cortical bone was simulated by means of a layer of glass-fiber-reinforced resin, while the spongy bone was simulated by a core of unsaturated polyester. The experimental specimens were investigated under different experimental conditions; the main mechanical test was the three-point bending test.

## Materials and Methods

Twenty titanium fixtures were screwed in 10 resin mandible section simulators to mimic osseointegrated implants in the first premolar area and molar area. The screwed implants were then divided into two groups:

Group A: five Fixed Partial Dentures (FPDs) in zirconia-ceramic connected to titanium abutments.

Group B: five FPDs in PEEK-composite connected to titanium abutments.

All the tested samples were analyzed by micro-CT after the mechanical loadings.

### Creation of a bio-faithful mandibular section simulator (MSS)

The mandibular section simulator (MSS) used in this study was created according to the prototype described by Apicella et al. 28. To test the FPDs, we decided to use this bio-faithful system so that the experimental conditions were able to reproduce a clinical environment. According to the study of Schwartz-Dabney and Dechow 29 we created a model of resin mandible able to recreate a non-dentate section.

### Screwing of implants

**Figure 1:**
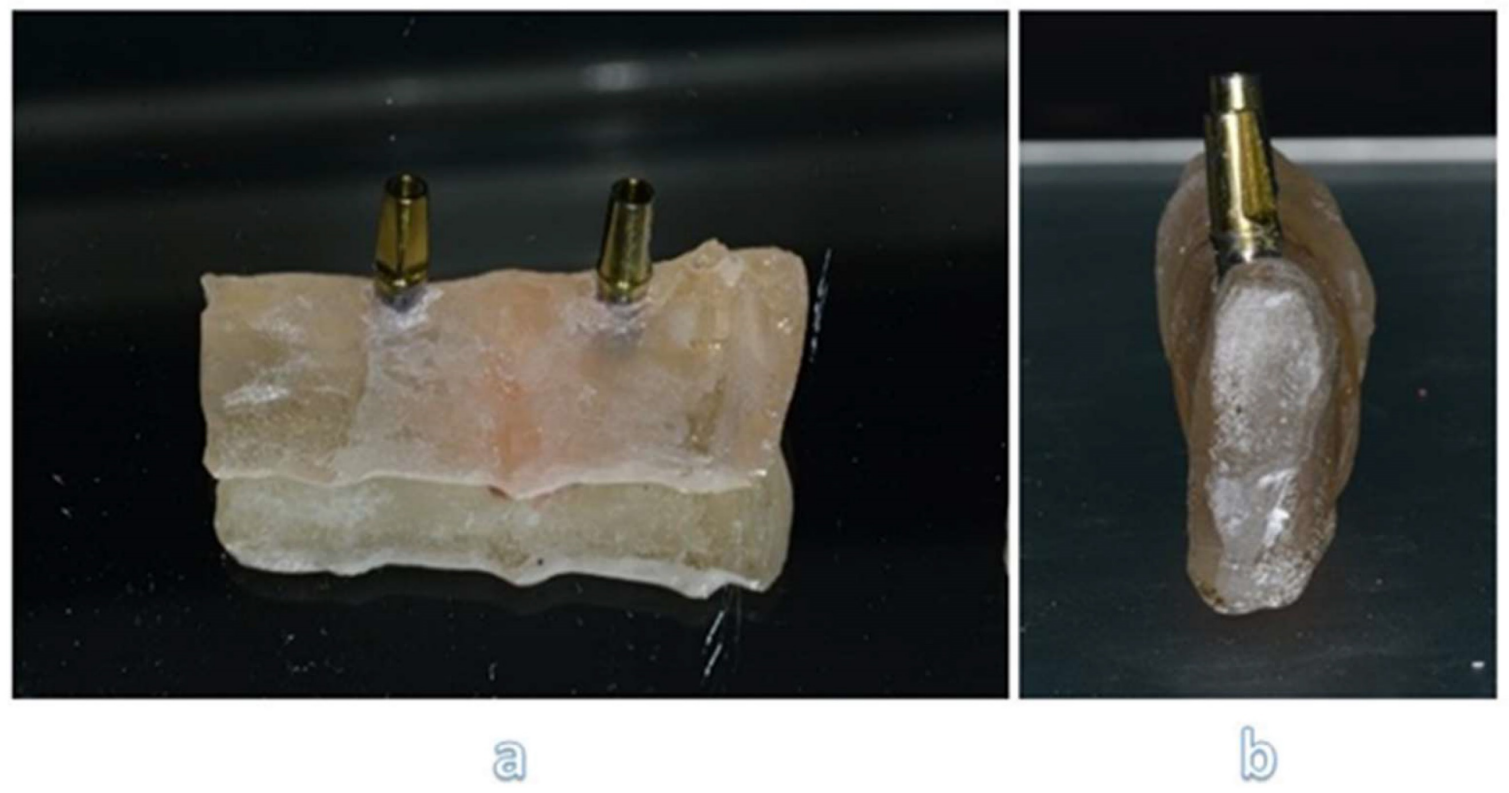
Two different perspectives of mandibular section simulator with two implants screwed inside: frontal view (a) and sagittal view (b).

After the creation of mandible model, we located two implants for each sample with inter-implant distance of 2,2 cm, to recreate a FPD with three elements: a first mandible premolar, a first mandible molar and a second mandible molar (**Figure 1 a, b**).

**Figure 2:**
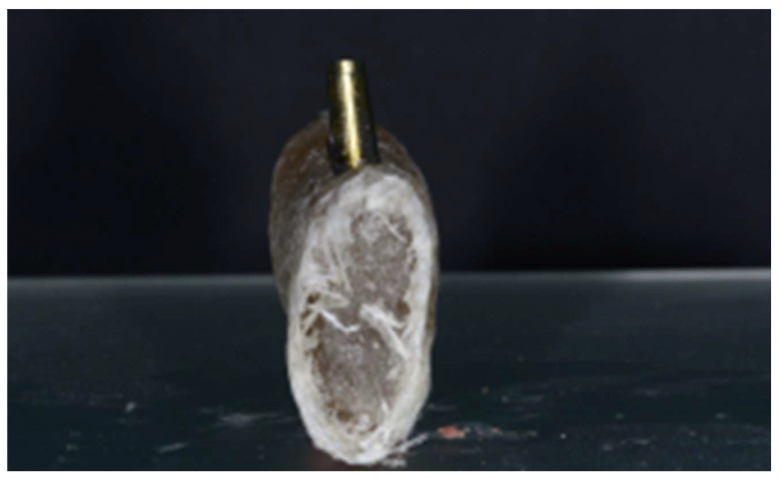
Section of sample with reinforced fiber.

We placed implants with the same procedures performed during a traditional in-vivo dental surgery. Upon each implant we screw two standard abutments, with a torque of 25 Ncm (**Figure 2**).

### Creation of FPDs models

**Figure 3:**
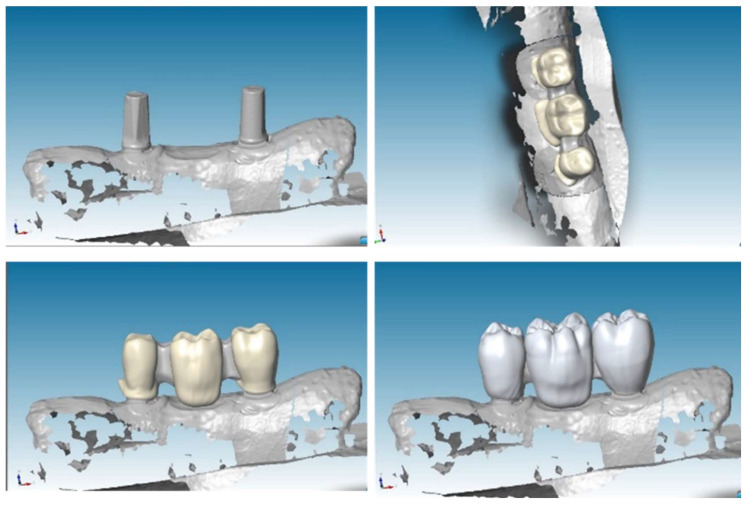
CAD project of frameworks: different wall thicknesses were designed for each CAD-CAM (Computer-Aided Design-Computer Aided Manufacturing) model.

To create a virtual model and design prosthesis and framework with specifical parameters, we scanned mandibular section using Dental Wings Software. Then, we improved the kinematic analysis with the virtual articulator; the precise occlusion simulation significantly reduces the time required for chairside occlusion adjustment, facilitating the overall procedures. With the CAD (Computer-Aided Design) model we created two frameworks: Zirconia and PEEK with the three elements above cited (**Figure 3**).

#### Zirconia (framework) ceramic (veneer)

**Figure 4:**
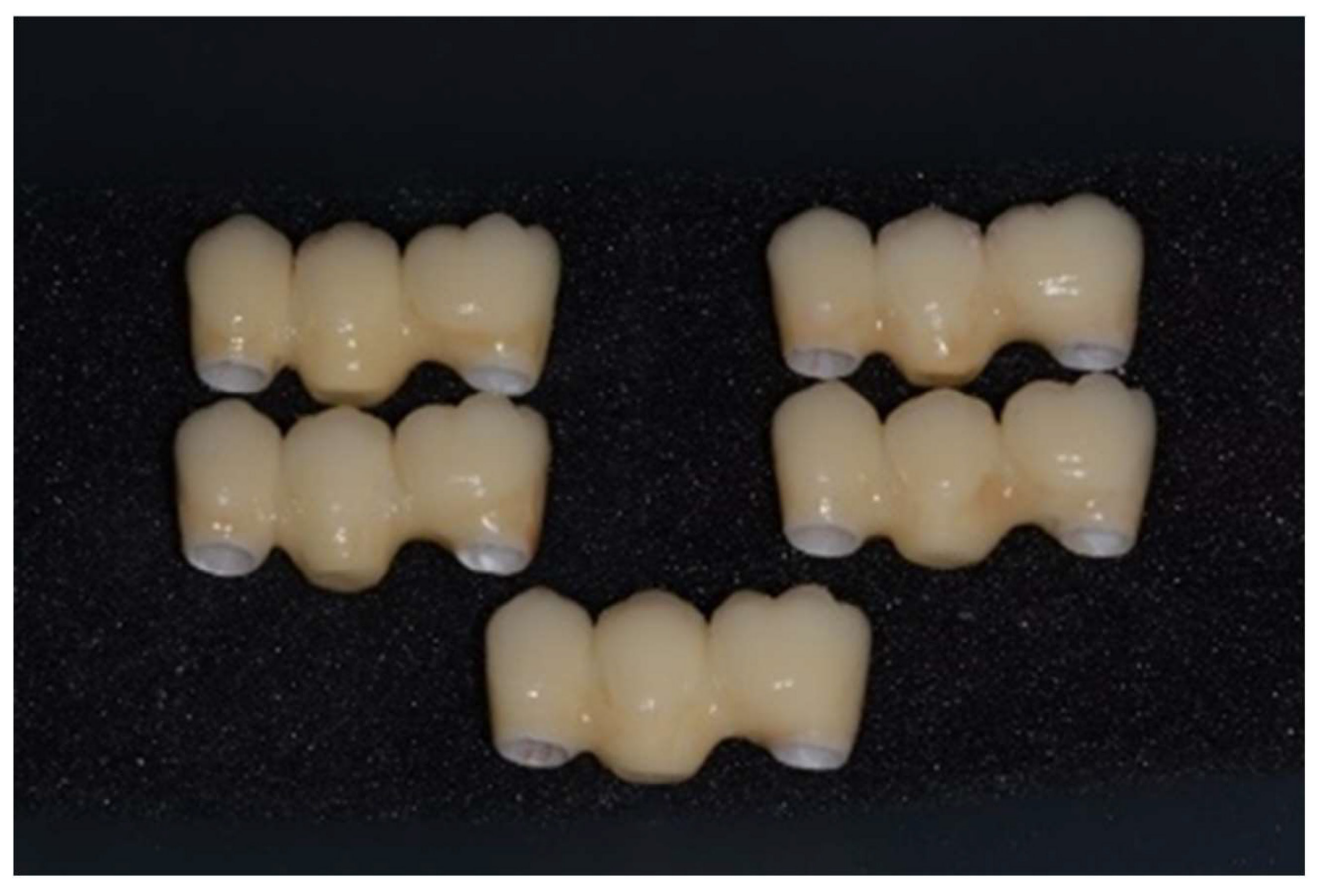
Samples with zirconia framework and ceramic rich in leucite veneer.

After getting CAD project, we have provided data to perform the CAM stage. In this study, we used pre-sintered colored discs (A2) of Zirconia multi-layered (Bluzirkon®Simex) which requires a further sintering process. In details, yttria-stabilized tetragonal zirconia polycrystal (3Y-TZP) has been used. To obtain the best mechanical performances by the prosthesis, we used a specific sintering protocol 4, performed as follows: the samples were sintered in an induction furnace (SpeedFire furnace, Dentsplay Sirona) for 18 minutes using a custom-programmed high-speed sintering cycle (Temperature (T) degrees: T1 = environmental temperature, T2 = 1400°, T3 = 1500°C, T4 = 1560°, T5 = 800°C). In this process the size of the prosthetic structures decreases by about 20%. After sintering, to evaluate the marginal fitting, the specimens are subjected to manual finishing. Then they were sandblasted with Al_2_O_3_ to 50μm, 2.5 Bar at 3-5 cm. After this process, the specimens are exposed to ceramic coating, characterized by a first layer of dentin and a final polish. In details, a leucite-based glass-ceramic (ceramic Noritake® ex-3) has been used for veneering. The specimens are then subjected to three cycles of thermal treatments (Tables 1-3) in a ceramic oven (Dentsply Sirona): firstly, a layer of veneering ceramic was fired, and a separable steel mold was used to layer the ceramic; ceramic powder was mixed with an appropriate amount of liquid, accordingly to the common practice in a dental laboratory, and filled into the silicon molding. In a second thermal treatment, an artificial dentin was added to compensate the shrinkage of the sintering process. Then, an additional heat treatment after sandblasting was performed: specimens were heated for 15 minutes at 1000℃ in the ceramic oven 30. Finally, the samples were polished with fine grit diamond mills (**Figure 4**).

#### PEEK (framework) composite (veneer) FPDs

After having obtained CAD project, we provided for data transfer to realize the implementation of CAM. In this study we used PEEK (VICTREZ ® PEEK)). After milling, the specimens are subjected to sandblasting with Al_2_O_3_ to 50μm for 45 seconds at 0.2 MPa, at a 45-degree angle, from 10 mm, and subsequently cleaned in an ultrasonic bath with distilled water for 5 minutes; pre-treatment was conducted with PEEK applied and light polymerized at 220 mW/cm^2^ for 90 seconds. To recreate with composite the exact veneers form, a silicone mold with zirconia-ceramic FPDs' volumes has been prefabricated on PEEK. After the application of veneering composite resin on the PEEK framework, the silicone molding was superimposed, and excess material was removed. Sinfony 3M® was the composite used in this study.

**Figure 5:**
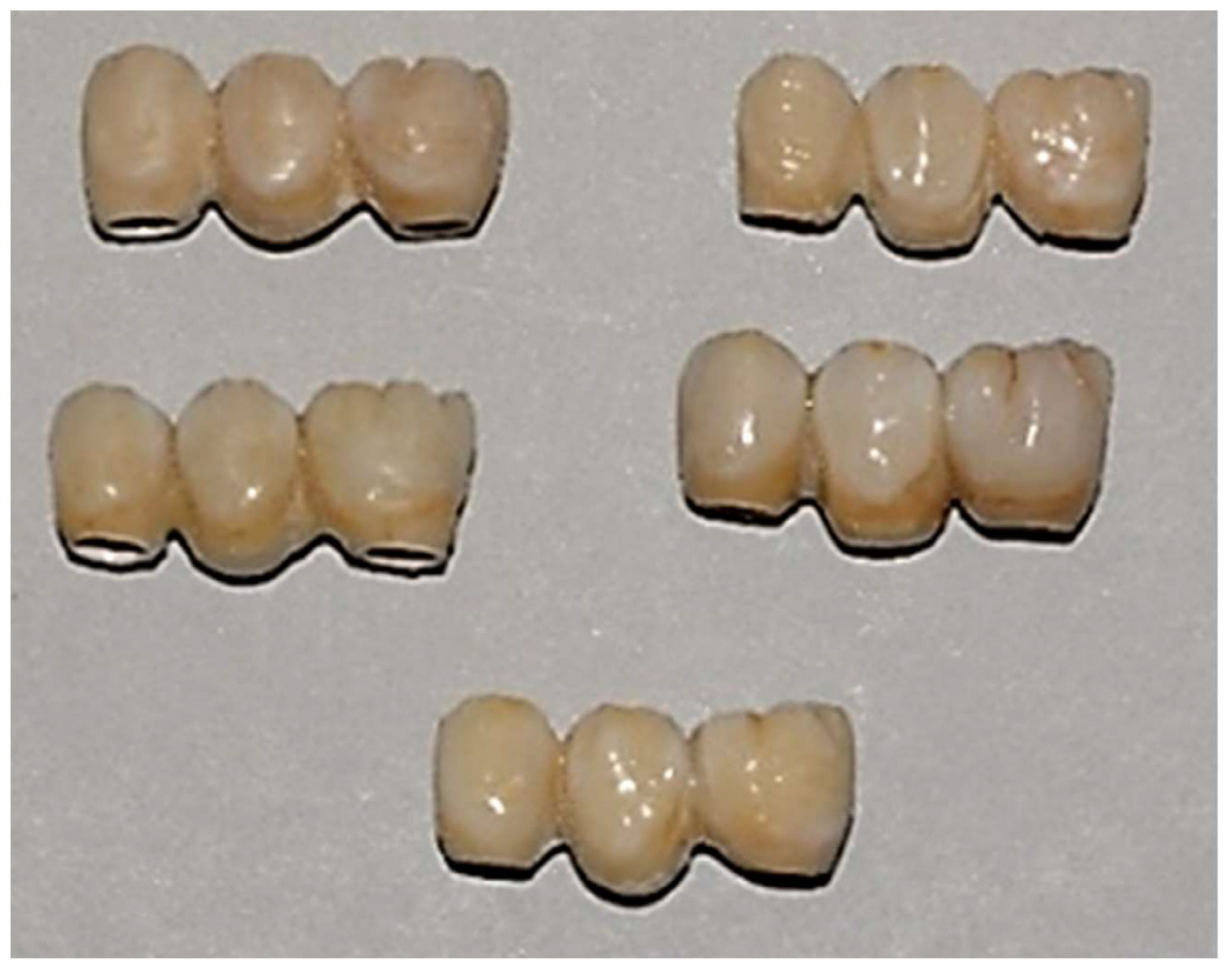
Samples with PEEK framework and resin composite veneering.

Finally, the specimens are polished with fine grit diamond mills. This process has been conducted to create high-luster restoration that offer an improvement in aesthetics (**Figure 5**).

### Three-point bending test

In our preliminary investigations, different methods were carried out before choosing to use the three-point bending test. The methods to induce prosthetic failure, or a material fracture, can be the followings: a. three-point flexure test (based on a nonuniform central stress field), b. four-point flexure test (based on a uniform central stress field), and c. biaxial flexure test. Many traditional mechanical tests are not suitable to our study, because they are not able to promote the conditions for the prosthetic failure in our specimens. In this landscape, the three-point bending test has been considered to be the proper method able to induce the highest tensile strength, thus, able to induce the most desirable stress to be applied to our samples. Therefore, the Fixed Partial Dentures have been analyzed according to three-point-bending method. The “metal-ceramic bond strength” test and the “three-point bending” test (min. 25 MPa) were performed according to EN ISO 9693.

Load was applied at the exact center of the structures, more precisely in the central connector (second mandible premolar) and in the lower constraints constituted by the implant structures.

To recreate a first-class occlusion, we provided a fusion of a second maxillary premolar. Then it was welded in the middle of roller bar. Before testing each sample, occlusion was calibrated.

The cyclic load applied to the structure ranges from a minimum of 0 N to a maximum of 860 N. Applying these values ​​to the equation [1N = 0.102 kg], we can say that our samples have undergone a load varying from 0 to 86,7 kg. This load is not random but responds to the parameters of a molar during the chewing cycle (75-89 kg) 31. The cyclical and incremental application of the load tends to simulate an act of mastication.

During testing phases extremities of mandible section must be fitted in a vise: in this way every deformation could be happened during test, but nevertheless the bending machine didn't push it during action.

More in details, this work aims to get reliable and comparable data, in order to analyze our different samples and materials. To do that, we needed to make some technical simplifications about the in-vitro dynamics occurring to our specimens investigated with the three-point bending test, differently to what happens in the in-vivo conditions: we considered the system as a “simply supported beam” (SSB) with a point load at center, thus, a not-distributed load. We overlooked both the bending moments and the shear forces that occur in the in-vivo conditions, following a distributed load. According to the following formula, “deflection calculation of a supported beam with central concentrated load”, we assumed that the stress is only due to the axial force.



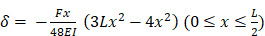



In the above equation: *δ*=deflection (mm); *F*=concentrated load (N); *E*=Young's modulus (MPa); *I*=moment of inertia (mm^4^); *L*=length of specimen (mm).

**Figure 6:**
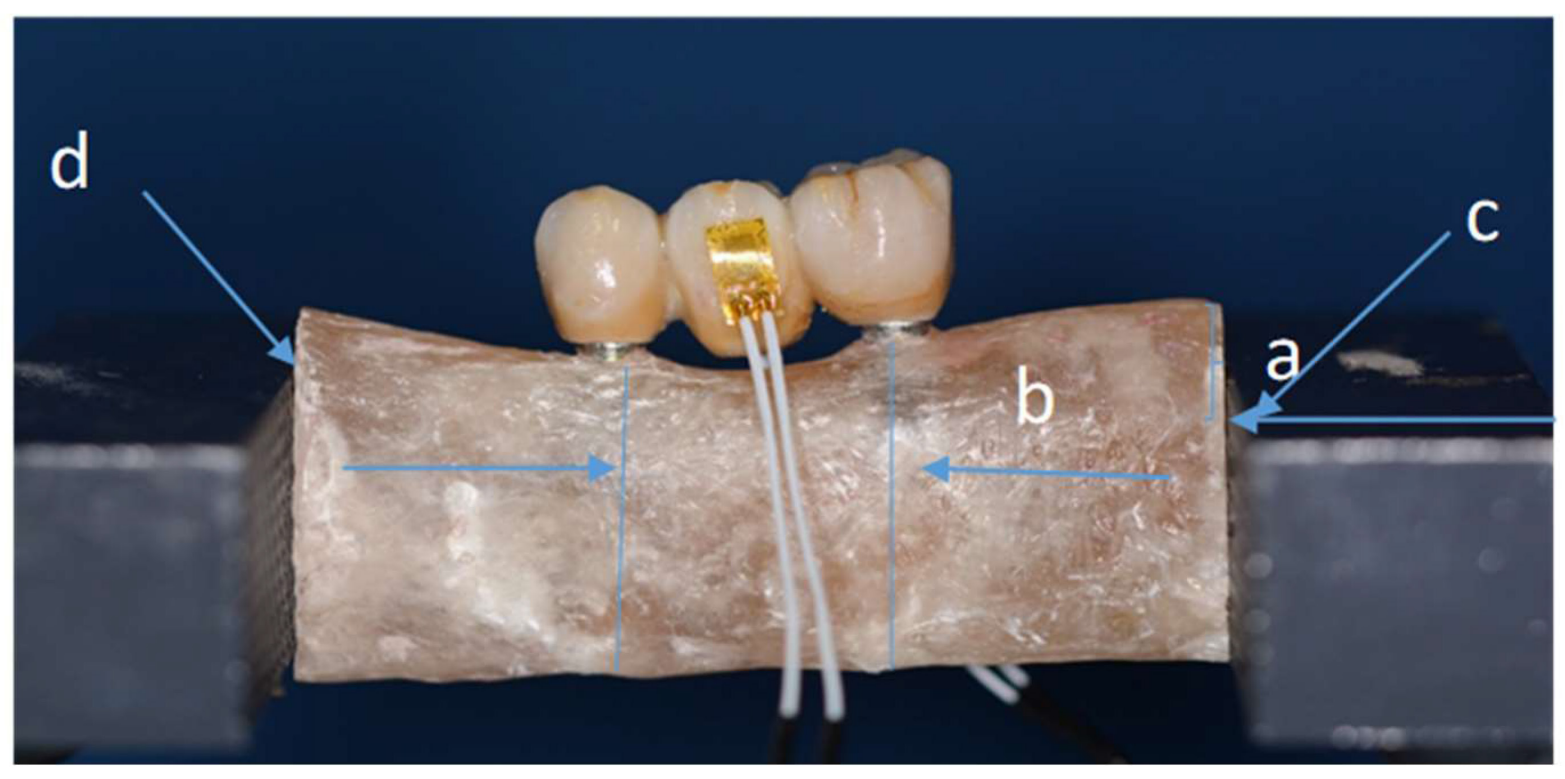
Sample fitted in a vise and with strain gauge connected. (Distance a-0.6 cm/ distance b-2 cm).

Every distance has been calculated and different stability tests have been done. e marked each specimen on right and left sides at points c-d, to make sure we don't have movement during tests (**Figure 6**).

**Figure 7:**
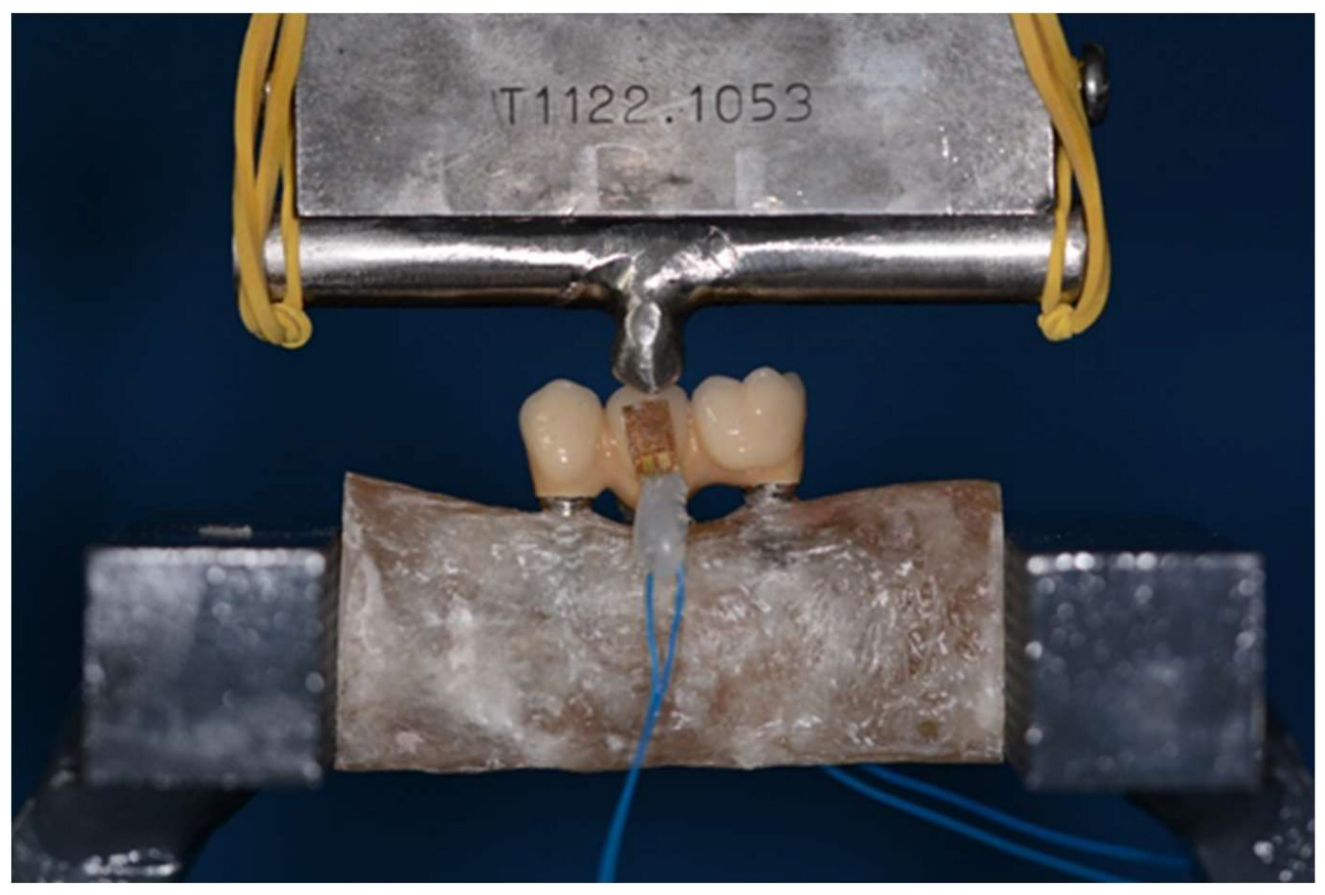
Sample during three-point bending test: load was applied at the exact center of the structure. The three-point-bending test was performed using a universal testing machine (Instron 5566®, UK) at a feed speed of 1 mm/min.

The three-point bending test was performed using a universal testing machine (Instron 5566®, UK) at a feed speed of 1 mm/min (**Figure 7**). For each material (zirconia-ceramic and peek) five three-point bending test with strain gauge machine connected have been conducted.

A linear strain-gauge self-compensated in temperature (C-980204, Micro-oup, Inc. Raleigh, North Carolina, USA) was bonded (Histoacryl, Braun, Italy) on the buccal and lingual aspect of FPDs. Strain-gauge was connected to a digital strain measuring hardware (Omicron-T, Battipaglia, Italy) interfaced to a personal computer equipped with a software providing data visualization and storage. The strain state was recorded as a function of time. The strain-gauge signals were recorded in the compressive modalities. This idea has been derived from literature 32.

### Micro-CT analysis

After mechanical loading, the samples were analyzed with a micro-computed cone-beam X-ray system (Skyscan 1072m-Ct, Kartuizersweg 3B, 2500 Kontich Belgium) without addition of contrasting agent. Image reconstruction and analysis were conducted using the software package provided by Skyscan, based on the Feldkamp algorithm 33. Images in TIFF format, and transversal sections of the sample in Bmp format can be obtained.

The sample were scanned at magnification of 15 X corresponding to 19,14 mm/pixel size resolution and with following setting: 100kV, 98 mA and using the 1 mm -Al filter. The data sets were acquired over rotation range of 180° (with 0.45° rotation step) and 3D reconstructed with a software (NRecon v1.6.10; Skyscan).

## Results

### Load-displacement functions

Bending results of each material (specimens=5) are represented on a graphic, where the x-coordinate data are related to feed speed (mm/min), that represents the speed at which the tool moves during the machining in the feed direction, while the y-coordinate are related to the load (N) applied.

#### Zirconia-ceramic composite FPDs

**Figure 8:**
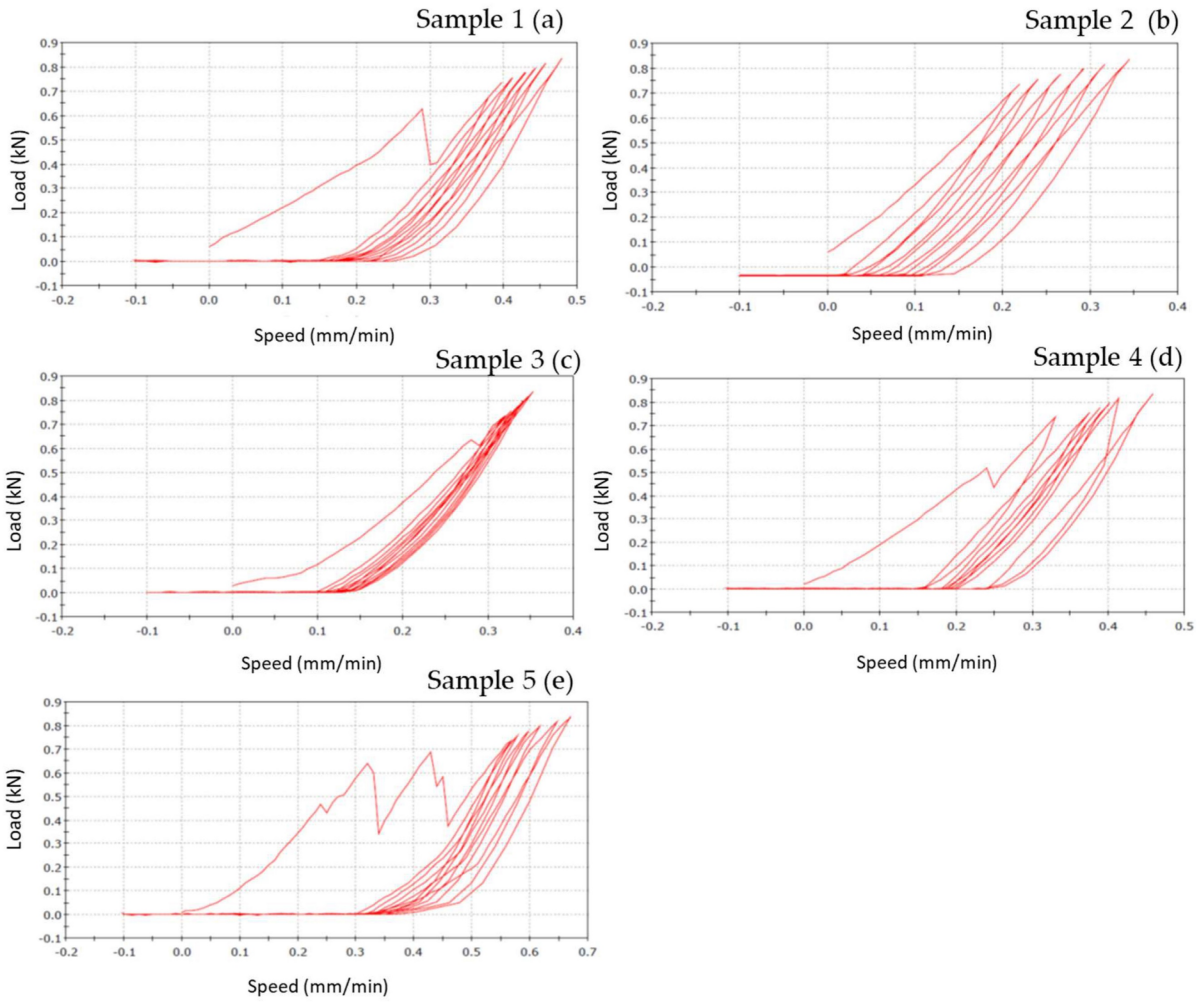
Load-displacement graphics of five zirconia specimens: the figure shows the vertical displacement of zirconia-ceramic FDPs related to the applied loads. In the graphics, x-coordinate data are related to speed (mm/min), while y-coordinate are related to load (N).

Zirconia-Ceramic FPDs load-displacement graphics shows that sample n. 1 presents chipping around the apical part of structure, in premolar and molar areas (**Figure 8 a**). The outcome of sample n. 2 is chipping around the apical part of structure, in premolar and molar areas, and fracture on the lingual side of first premolar (**Figure 8 b**). Sample n. 3 shows chipping around the apical part of structure, in premolar and molar areas, occlusal fracture and fracture of first premolar in mesial-distal area (**Figure 8 c**). Sample n. 4 presents chipping around the apical part of structure, in premolar and molar areas, fracture on the lingual side of first premolar and framework's fracture around the junction abutment-zirconia (**Figure 8 d**). Lastly, sample n. 5 shows a total compound fracture along the first premolar pontic connection (fracture load-735 N) (**Figure 8 e**).

Figure 8 shows the vertical displacements of zirconia-ceramic FDP as a function of the applied loads. In all tested samples the unloading ramp didn't recover the starting point of the loading ramp. Consequently, the successive loading ramp started with more vertical displacements with respect to the initial loading ramp. The same behavior was noticed for the subsequent loading and un-loading ramps. After 7 cycles the loading ramp started from a more vertical displaced point. The vertical permanent displacement of the zirconia samples was 90 (+/- 22) μm. Such behavior was noticed in all zirconia ceramic tested samples except for sample 3 where the vertical displacement starting point was constant trough out the entire test. The first derivative of the functions remains constant throughout the tests.

#### PEEK-composite FPDs

**Figure 9:**
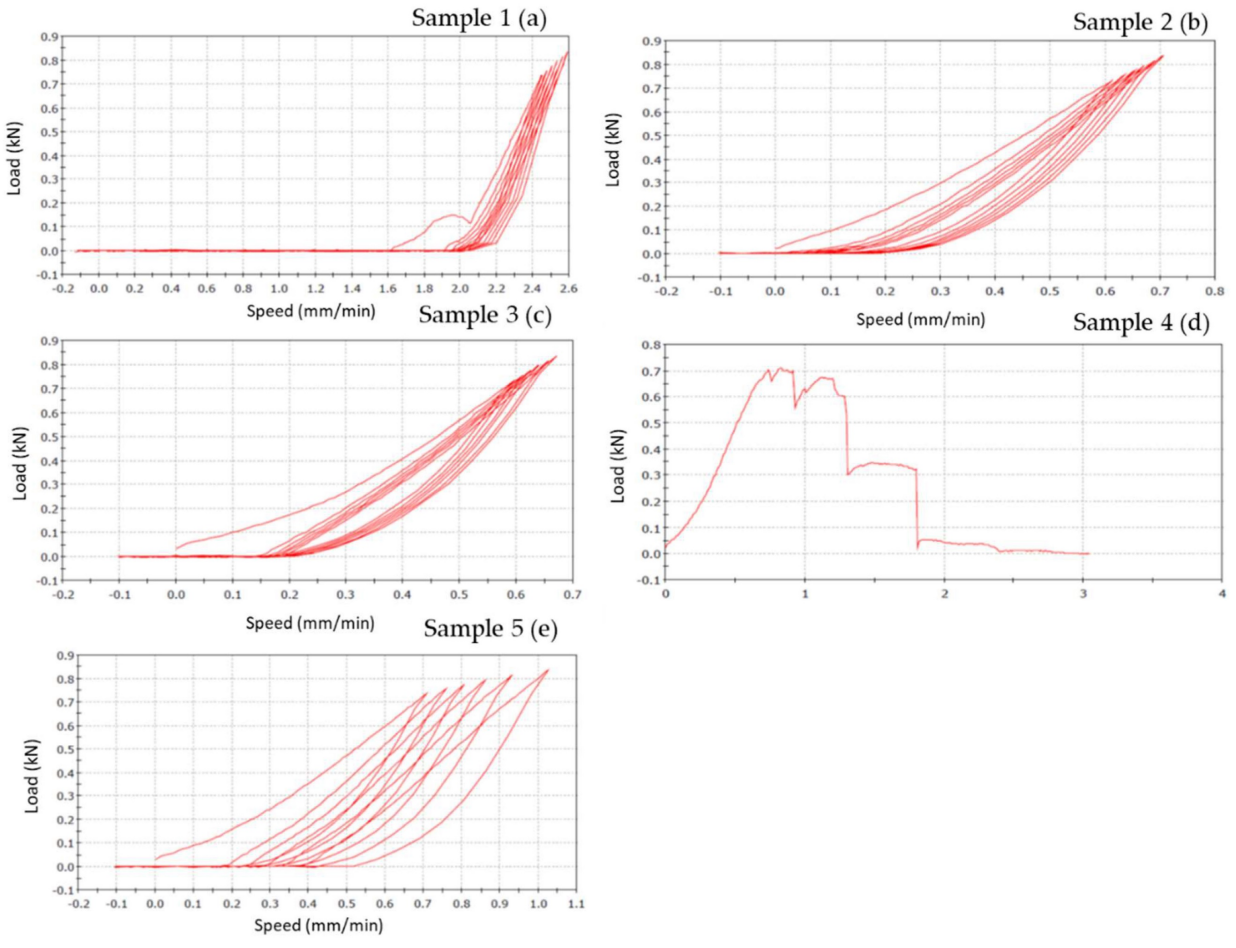
Load-displacement graphics of five PEEK specimens: the figure shows the vertical displacement of PEEK-composite FDPs related to the applied loads. In the graphics, x-coordinate data are related to speed (mm/min), while y-coordinate are related to load (N).

PEEK-composite FPDs load-displacement graphics show that sample n.1 presents occlusal chipping in second premolar area (**Figure 9 a**) while sample n. 2 presents mesial-lingual crack in molar area and disto-buccal crack in second premolar area (**Figure 9 b**). Sample n. 3 shows thin chipping in the apical part of second premolar and first molar (**Figure 9 c**). The outcome of specimen n. 4 is a total fracture between the first premolar and the second premolar (fracture load 700 N) (**Figure 9 d**). Samples n. 5 shows superficial thin crack in second premolar area (**Figure 9 e**).

However, the graph of sample n.1 (**Figure 9 a**) shows a different slope respect to other samples. This fact is due to the sample slipping from the base during the initial phase of the test.

**Figure 9** shows the vertical displacements of PEEK-composite FDPs as a function of the applied loads. In the PEEK-composite samples, the horizontal offset of the curves, due to the permanent vertical displacements, was of 0.4 mm and thus, significantly higher than that the zirconia specimens.

The following table (**Table 4**) shows the values of vertical displacement obtained from each case study.

### Mechanical test analysis

**Figure 10:**
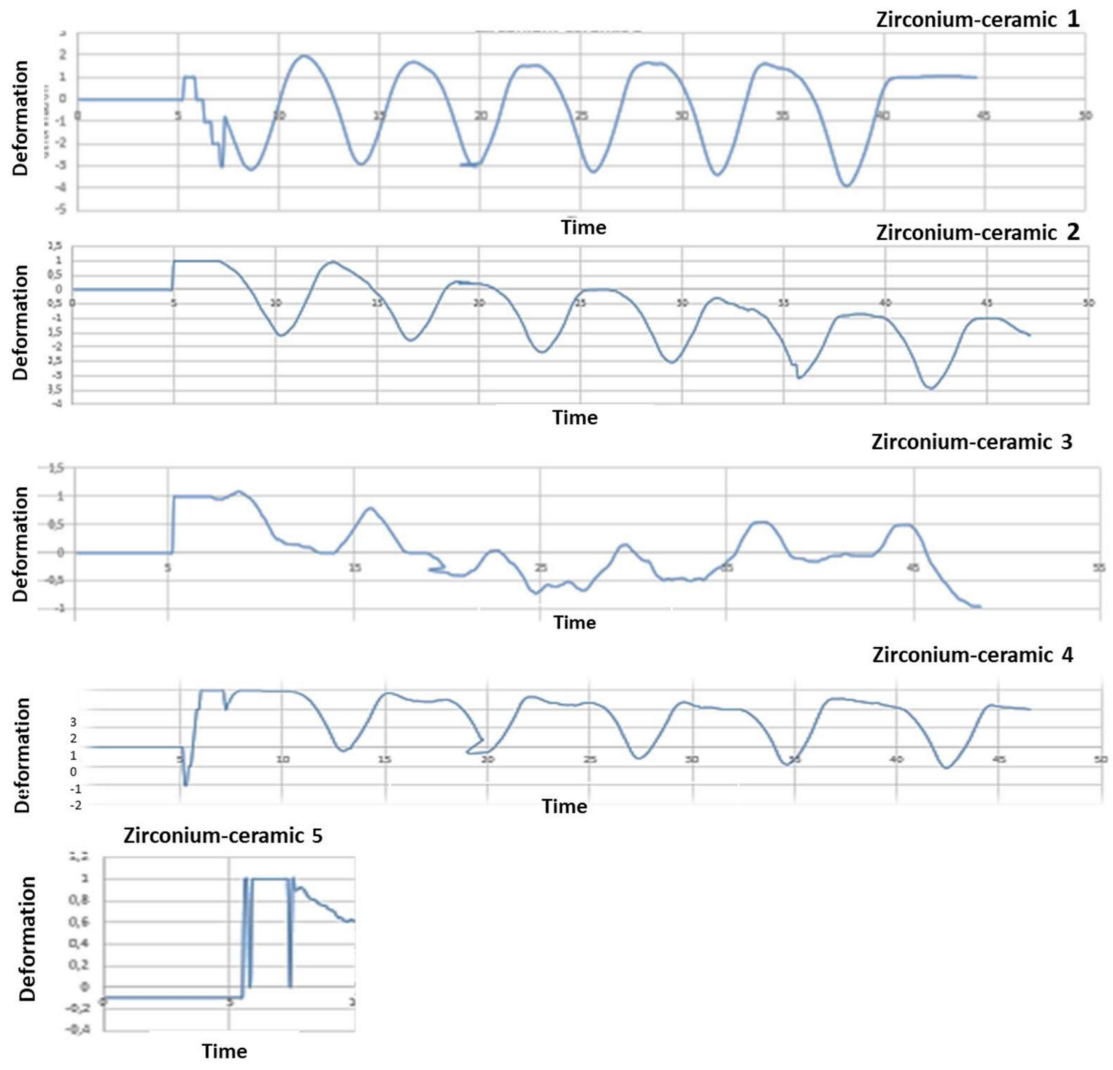
Five zirconia-ceramic samples strain gauge graphics. The figure shows strain gauge graphics obtained to strain gauge machine. In the graphics, x-coordinate data are related to time (s), while y-coordinate data are related to deformation E.

After mechanical test, strain gauge graphic obtained to strain gauge machine has been converted in a numerical series. A graphical representation with time like x-parameter and y-parameter deformation has been obtained: time/deformation (s/E). **Figure 10** (zirconia-ceramic) shows the strain-time for all tested samples. The application of 750 N produced a compressive strain state of 1,5 E-05 (+/-5,7E-06), while the application of 850 N produced a compressive strain state of 1,8E-05 (+/-1,4E-07).

**Figure 11:**
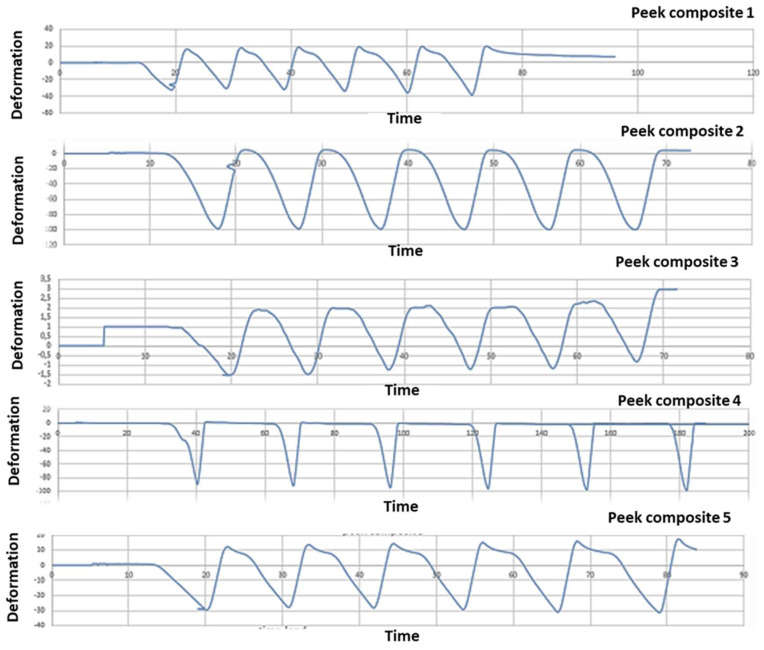
Five PEEK- composite samples strain gauge graphics. The figure shows strain gauge graphics obtained to strain gauge machine. In the graphics, x-coordinate data are related to time (s), while y-coordinate data are related to deformation E. The variability between the results shown in the **graph 10** and **11** is due to heterogeneity between the samples. The load has been the same to all the samples investigated; nevertheless, the manual finishing of samples has affected the homogeneity of the samples. Moreover, although a silicone mold has been used to homogenize the samples, unfortunately, the same silicon mold was another technical reason of the structural defects.

PEEK-composite FDP underwent an average compressive strain of 0,00032 (+/- 1,3E-05) (**Figure 11**).

### Micro-CT analysis

**Figure 12:**
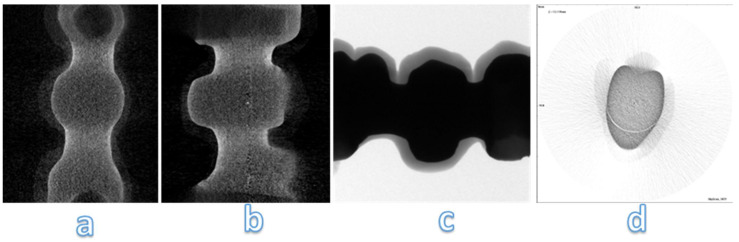
Micro-CT analysis of zirconia-ceramic specimens. The samples were analyzed with a micro-computed cone-beam x ray system and were scanned at magnification of 15 X. The figure shows different sections of specimens: occlusal section (a) and sagittal sections (b, c, d)

From the analysis of the images obtained at the micro-CT is impossible to detect structural defects inside the frameworks of zirconia (Figure 12). Despite zirconia-ceramic has been the worst sample that we have tested, no cracks are visible. High density of framework doesn't allow to underline structural defects.

**Figure 13:**
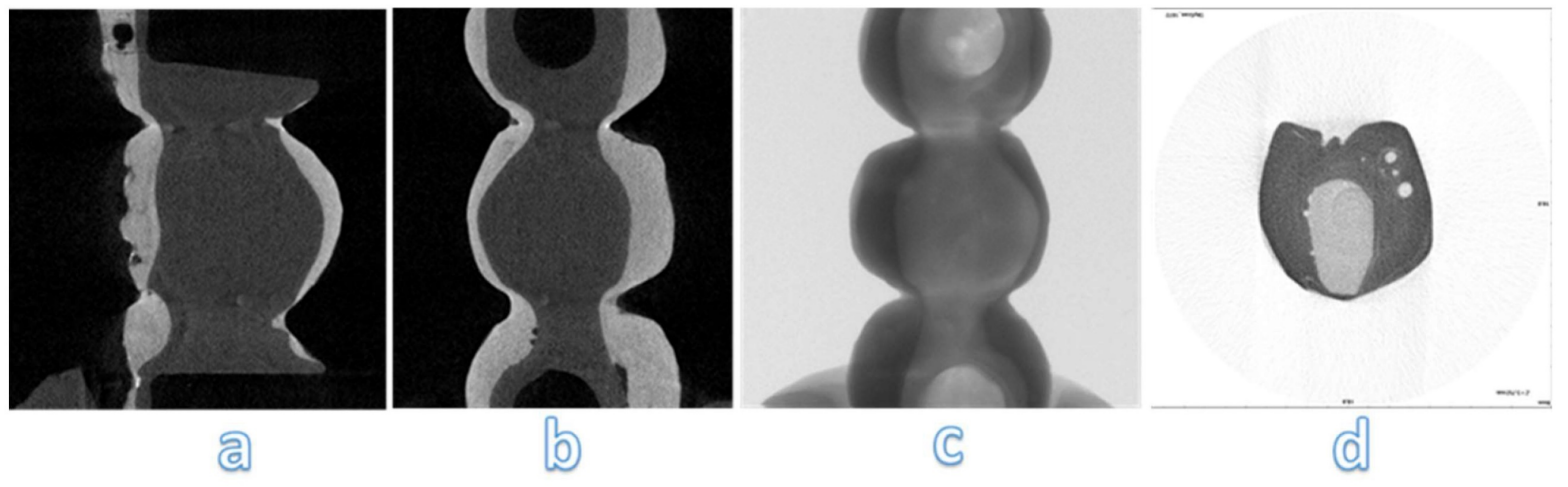
Micro-CT analysis of PEEK-composite specimens. The samples were analyzed with a micro-computed cone-beam x ray system and were scanned at magnification of 15 X. The figure shows different sections of specimens: occlusal section (b, c) and sagittal sections (a, d)

Analysing images obtained at micro-CT, structural defects are noted inside PEEK-composite veneering (Figure 13 a, b, c). Spherical air cells are present in the inner part of the framework and scattered in composite veneering; however, cracks aren't detected near bubble (Figure 13 d). Silicone mold used to obtain similar samples has been the real trouble of these structural defects.

## Discussion

This study has compared the load-displacements of two different materials following three-point bending test created with the CAD-CAM technique. Previously study have demonstrated that zirconia FDPs group obtained a lowest load to fracture values respect to PEEK group 34. In the present study these data are confirmed. In zirconia composite the constant first derivative of the load-displacement functions indicate that the component rigidity is not decreased by the test. Nevertheless, after each mechanical test an unrecovered vertical displacement is noticed. It could be hypothesized that this result is due to the loss of integrity of the zirconia ceramic FPD in the connection areas to the abutments as well as a possible permanent deformation of the titanium abutments. The visual analysis of failed samples suggested that the above mentioned unrecovered vertical displacement is due to the chipping phenomenon observed in the FPD where it is connected to the abutments. Although the chipping occurring, the constant first derivative of the loading-displacements functions suggests that the FDP maintained his elasticity and rigidity.

In the analysis of FPDs, previously conducted by Cardelli et al. 37, stresses are mainly concentrated on the surface where the load is applied; in fact, it is much more rigid, and close to the edge of the mesial abutment. The concentration of considerable stresses on such surfaces can be directly correlated to the chipping, which is usually found in the distal margins of the structure under investigation.

Another important aspect is the full strain state recovery after each loading cycles. Despite strain gauges were positioned on the FDPs central unit they were not influenced by the chipping. The full strain recovery indicated a full elastic behavior of the central unit. The vertical permanent displacement observed in PEEK-composite samples can be explained by the plastic deformation of the PEEK structure followed by the plastic deformation of the resinous composite matrix. In all tested samples the strain gauge analysis showed a linear correlation between the applied loads and recorded strain. Such fact indicates that the loss of integrity of the FDP close to the abutments didn't not reduce the rigidity of the system. A full recovery of undeformed shape was observed in all tested samples.

During the testing of zirconia-ceramic samples, the surface fractures occur at 650 N 28. In our study, the structure has been tested under an extreme condition: the incremental load upon the intermediate pontic-element. This is a condition complex to be managed and recovered in a clinical setting.

The PEEK composite groups show a compressive strain state twenty time higher than the zirconia ceramic; these data confirmed those already noted in literature 34. The rigidity of the zirconia ceramic FDP is significantly higher respect to the PEEK-composite. Furthermore, in the PEEK-composite, the higher rigidity contribution is ensured by the composite veneer.

PEEK-composite samples showed a typical viscous failure pattern while a brittle failure pattern was observed in the composite volumes. The low Young's modulus ofPEEK makes it as elastic as bone: this property allows it to act as a stress breaker, and consequently allows it to reduce the forces working on the tooth roots. 35,36. In fact, DalPiva et al. reported a low elastic modulus on PEEK crowns compared to other materials, including zirconia 38.

The present study was conducted *in vitro* and under standardized conditions. However, some studies used resin abutments 39, while other studies used metal abutments 40. Furthermore, in the present study, real frameworks have been used while disc or cylinder specimens have been used in other works 41. These differences can lead to discrepancies between different studies using the same materials.

According to other studies, in our work only the static load has been used, thus deeming adequate the compressive forces for evaluating the fracture resistance on FPDs 42. Otherwise, several authors included artificial aging to reproduce the oral environment 43.

The main limitation of this study is the lack of in-depth statistical analyses, including the calculation of means and standard deviation; moreover, the high variability among samples, and the lack of exposition to aging conditions of the samples may require in future further investigations. This research could be also improved with a better characterization of samples at baseline; further investigation will aim to verify implant stress distribution after this type of loads, and to compare the mechanical results with FEA/FEM analysis.

## Conclusions

For selecting the restorative biomaterials used for fixed prostheses, mechanical properties of these materials are primary criteria. In this study two different materials, Zirconia and PEEK, have been manufactured by CAD-CAM technique to fabricate FPDs. These *in vitro* studies have compared the load to fracture of the above-mentioned materials by three-point bending test. From this study, different conclusions were drawn: the type of material influenced the load to fracture; for the tested samples, different fractures have been observed; PEEK could be an alternative to ceramic o metal materials.

In detail,* in vitro* tests of this study have shown that PEEK could be considered a good material with a high value of vertical displacement and a great elastic deformation. The small difference between E-modulus of two materials (5 GPa PEEK/3 GPa Sinfony) improve its physical and mechanical characteristics.

PEEK exhibited the highest load to fracture values. Therefore, PEEK could be considered a suitable alternative to metal materials for prosthetic solutions. However, clinical investigations will be needed to overcome the limitations of *in vitro* study, though important aspects of clinical environment were simulated.

## Figures and Tables

**Table 1:** First thermal treatment values

Low temperature	600°	Vacuum level	-740 mmHg
**Rising time**	06:00	**High temperature**	945°
**Preheating time**	02:00	**Maintenance temperature**	
**Degrees/min**	45°/min	**Final temperature**	945°
**Departure/vacuum**	600°		
**End vacuum**	895°	**Down time**	02:00

**Table 2:** Second thermal treatment values

Low temperature	600°C	Vacuum level	-740mmHg
**Rising time**	10:00	**High temperature**	940°C
**Preheating time**	02:00	**Maintenance temperature**	
**Degrees / min**	45°C/min	**Final temperature**	940°C
**Departure /vacuum**	600°C		
**End vacuum**	900°C	**Down time**	04:00

**Table 3:** Third thermal treatment values

Low temperature	600	Vacuum level	
**Rising time**	02:00	**High temperature**	940°C
**Preheating time**	01:00	**Maintenance temperature**	
**Degrees / min**	46°C/min	** Final temperature**	940°C
**Departure /vacuum**			
**End vacuum**		**Down time**	02:00

**Table 4:** Vertical permanent displacement (VPD) obtained in PEEK and zirconia specimens.

Samples	VPD
Zirconia-ceramic	90 (+/- 22) μm
PEEK-composite	0.4 mm
